# Definition of factors associated with negative antibody response after COVID-19 vaccination in patients with hematological diseases

**DOI:** 10.1007/s00277-022-04866-z

**Published:** 2022-05-21

**Authors:** Jil Rotterdam, Margot Thiaucourt, Christel Weiss, Juliana Schwaab, Andreas Reiter, Sebastian Kreil, Laurenz Steiner, Sebastian Fenchel, Henning D. Popp, Wolf-Karsten Hofmann, Karin Bonatz, Catharina Gerhards, Michael Neumaier, Stefan A. Klein, Sonika Rao, Mohamad Jawhar, Susanne Saussele

**Affiliations:** 1 Department of Hematology and Oncology, III. Medical Clinic, University Hospital Mannheim, Heidelberg University, Theodor-Kutzer-Ufer 1-3, 68167 Mannheim, Germany; 2 Institute for Clinical Chemistry, University Hospital Mannheim, Heidelberg University, Mannheim, Germany; 3 Department of Medical Statistics, Biomathematics and Information Processing, Center for Preventive Medicine and Digital Health Baden-Württemberg (CPD-BW), University Hospital Mannheim, Heidelberg University, Mannheim, Germany

**Keywords:** SARS-CoV-2, COVID-19 vaccination, Hematological diseases/disorders, Seroconversion

## Abstract

COVID-19 in patients with hematological diseases is associated with a high mortality. Moreover, preventive vaccination demonstrated reduced efficacy and the knowledge on influencing factors is limited. In this single-center study, antibody levels of the SARS-CoV-2 spike protein were measured ≥ 2 weeks after 2nd COVID-19 vaccination with a concentration ≥ 0.8 U/mL considered positive. Between July and October 2021, in a total of 373 patients (median age 64 years, 44% women) with myeloid neoplasms (*n* = 214, 57%), lymphoid neoplasms (*n* = 124, *n* = 33%), and other diseases (*n* = 35, 10%), vaccination was performed with BNT162b2 (BioNTech), mRNA-1273 (Moderna), ChADOx1 (AstraZeneca), or a combination. A total of 229 patients (61%) were on active therapy within 3 months prior vaccination and 144 patients (39%) were previously treated or treatment naïve. Vaccination-related antibody response was negative in 56/373 patients (15%): in 39/124 patients with lymphoid neoplasms, 13/214 with myeloid neoplasms, and 4/35 with other diseases. Active treatment per se was not correlated with negative response. However, rituximab and BTK inhibitor treatment were correlated significantly with a negative vaccination response, whereas younger age and chronic myeloid leukemia (CML) disease were associated with positive response. In addition, 5 of 6 patients with myeloproliferative neoplasm (MPN) and negative vaccination response were on active treatment with ruxolitinib. In conclusion, a remarkable percentage of patients with hematological diseases had no response after 2nd COVID-19 vaccination. Multivariable analysis revealed important factors associated with response to vaccination. The results may serve as a guide for better protection and surveillance in this vulnerable patient cohort.

## Introduction

Patients with hematological diseases are at higher risk to develop severe coronavirus disease 2019 (COVID-19) caused by the severe acute respiratory syndrome coronavirus 2 (SARS-CoV-2). Studies demonstrated that COVID-19 is associated with a higher hospitalization rate, longer intensive care unit stays, and a higher mortality rate (up to 30%) compared to the general population [1–8].

In patients with hematological diseases, a compromised immune system may affect preventive vaccination as demonstrated by reduced efficacy not only for COVID-19 as several studies have shown [9–11]. Mortality rate of COVID-19 after vaccination in this patient cohort is still high with around 12% [12]. Patients with non-Hodgkin lymphoma (NHL), higher age, and immune-compromising B cell–depleting therapy are at risk for reduced vaccination response against COVID-19 [7, 9, 13, 14].

In addition, studies have shown a correlated time-dependent reduction in antibody titers over the time. Among persons of 60 years or older who became fully vaccinated in January 2021, the rate of breakthrough infections was 1.6 times higher compared to those who became fully vaccinated in March 2021 [15–17]. So far, little is known about this time-dependent reduction of antibody titers in patients with hematological disease as well as correlation to therapy and outcome after COVID-19 [8].

The aim of our study was to evaluate vaccination-related antibody response to BNT162b2 (BioNTech), mRNA-1273 (Moderna), and ChADOx1 (AstraZeneca) in patients with various hematological disorders and to identify prognostic factors influencing vaccination response.

## Materials/subjects and methods

### Subjects

In this observational single-center study, data were collected from July to October 2021. Patients with hematological diseases and a scheduled appointment were registered regarding their vaccination status. Patients who received the 2nd COVID-19 vaccination at least 2 weeks prior the appointment were included. As a control group, patients with benign and autoimmune diseases were also registered.

As part of routine patient care, 7.5-mL serum and heparin samples were collected. After adequate clotting of serum samples during 1 h at room temperature, samples were centrifuged at 2000 g for 10 min at 18 °C. Plasma was similarly centrifuged/separated and used for the subsequent analyses. A Food and Drug Administration/Continuing Education (FDA/CE)–approved electrochemiluminescent assay (ECLIA) (Elecsys®, Roche, Mannheim, Germany) was used to quantify serum antibodies, pan Ig (including IgG) against the receptor binding domain (RBD) of the SARS-CoV-2 spike protein. The assay has a measurement range of 0.4 to 250 U/mL, with a concentration ≥ 0.8 U/mL considered positive. Data were analyzed for patients without detection of anti-N (nucleocapsid) SARS-CoV-2 antibody. As it is unclear which level of antibody represents a sufficient protection, we here focus on the analysis of patients with negative antibody response vs. patients with any response [18, 19]. All tests were performed according to the manufacturer’s instructions in an accredited laboratory at the University Hospital Mannheim.

For analyses, patients were divided into disease subgroups. Additional patient data were collected: gender, age, therapy status (active treatment vs. previously treated defined as < vs. ≥ 3 months before vaccination), vaccination date, and vaccine type (BNT162b2, BioNTech/Pfizer; mRNA-1273, Moderna; ChADOx1, AstraZeneca Oxford).

### Statistical analyses

Baseline covariates were compared between vaccination responders and non-responders using either the Mann–Whitney *U* test, chi^2^ test, or Fisher’s exact test, as appropriate. Each of these tests has been carried out as a two-sided test. Univariable logistic regression analyses have been performed in order to calculate odds ratios for several factors. Furthermore, a multiple logistic regression analysis for the binary outcome “responder” has been performed in order to analyze several variables simultaneously. In general, the significance level was set to 0.05. All statistical calculations were performed with SAS (release 9.4, SAS Institute Inc., Cary, NC).

## Results

Overall, 373 patients with various hematological disorders were included in this study. The median age was 64 years with a range from 20 to 92. A total of 164/373 of patients (44%) were female, and 209/373 were male (56%). Vaccination was performed with BNT162b2 (BioNTech) (*n* = 289, 77%), mRNA-1273 (Moderna) (*n* = 36, 10%), ChADOx1 (AstraZeneca) (*n* = 26, 7%), or ChADOx1 first and BNT162b2 second (*n* = 22, 6%).

A malignant hematological disorder was diagnosed in 338/373 patients (91%) with myeloid neoplasms (*n* = 214, 57%) and lymphoid neoplasms (*n* = 124, 33%). Other diseases (*n* = 35, 9%) were autoimmune and benign diseases. Overall, 229 (61%) patients were on active antitumoral therapy, and 144 (39%) were previously treated or treatment naïve (see Table 1).

**Table 1:** Patients characteristics of total cohort and results in vaccination-related negative antibody response

Category	Total patient cohort (*n* = 373)		With positive antibody response (*n* = 317)		With negative antibody response (*n* = 56)			
	Number	Percentage	Number	Percentage of total group (% of 373)	Number	Percentage of neg. AB response (% of 56)	Percentage of subgroup	Percentage of total cohort (% of 373)
Gender, *n* (%)								
Female	164	44%	146	39%	18	32%	11%	4.8%
Male	209	56%	171	46%	38	67%	18%	10%
Age								
20–42	85	22%	85	23%	0	0%	0%	0%
43–64	116	31%	103	28%	13	23%	11%	3.5%
65–92	172	46%	129	35%	43	76%	25%	12%
Vaccination								
BNT162b2	289	77%	248	66%	41	73%	14%	11%
mRNA-1273	36	10%	26	7.0%	10	18%	28%	2.7%
ChaDOx1	26	7.0%	23	6.2%	3	5.4%	12%	0.8%
ChaDOx1 first. second BNT162b2	22	5.9%	20	5.4%	2	3.6%	9%	0.5%
Vaccination-related antibody response								
Positive	317	85%	317	100%	0	0%	0%	0%
Time between vaccination and analysis. mean (range): 9 (2–32) weeks								
Negative	56	15%	0	0%	56	100%	100%	15%
Time between vaccination and analysis. mean (range): 9 (2–28) weeks								
Hematological disease entities								
Malignant	338	91%	286	78%	52	93%	15%	14%
*Myeloid neoplasms*	*214*	*57%*	*201*	*54%*	*13*	*23%*	*6.1%*	*3.5%*
*Lymphoid neoplasm*	*124*	*33%*	*85*	*23%*	*39*	*70%*	*31%*	*10%*
Other	35	9.4%	31	8.3%	4	7.1%	11%	1.1%

Treatment included BCR-ABL tyrosine kinase inhibitors (TKI, *n* = 66), JAK2-inhibitors (ruxolitinib and fedratinib, *n* = 32), hydroxyurea (*n* = 21), steroids (*n* = 17), rituximab-based regimens (*n* = 13), interferon-alpha (*n* = 9), lenalidomide (*n* = 9), BTK inhibitors (ibrutinib and acalabrutinib, *n* = 8), immunoglobulin substitution (*n* = 7), hypomethylating agents and venetoclax (*n* = 7), any other chemotherapy (*n* = 7), and other tumor-specific therapies (*n* = 21).

Vaccination-related antibody response was positive in 317/373 patients (85%) with a median level of 197 U/mL (range 0.8–250 U/mL) and negative in 56/373 patients (15%). Of the patients with positive seroconversion, 63/317 patients (20%) had an antibody level between 0.8 and 100 U/mL. Mean time from vaccination to measurement was not different in both cohorts. The average analysis was after 9 weeks, with a minimum of 2 weeks (both groups) and a maximum of 28 weeks (group with negative seroconversion) and 32 weeks (group with positive seroconversion).

In selected patients, antibody titers were measured twice at 2 and 4 months after the 2nd COVID vaccination: A decrease from 250 to 171 U/mL was noted in one patient and from 66 to 5 U/mL in another. Thus, antibody titers in both patients decreased significantly, even though they are considered positive.

### Impact of disease type

The distribution of the negative response group regarding the disease subgroups was as follows: lymphoid neoplasms (39/56, 70%), myeloid neoplasms (13/56, 23%), and autoimmune diseases (4/56, 7%). In lymphoid neoplasms, patients with indolent NHL had the highest proportion of negative seroconversion with 64% (*n* = 25/39), followed by patients with aggressive NHL (21%, *n* = 8/39). In the group with positive response, percentages were 63% (lymphoid neoplasms), 27% (myeloid neoplasms), and 10% (autoimmune diseases). Overall, 69% (*n* = 85/124) of patients with lymphoid diseases had a positive vaccination response, while 31% (*n* = 39/124) showed no antibody response. In contrast, 94% (*n* = 201/214) of patients with myeloid diseases demonstrated seroconversion, whereas 6.1% (*n* = 13/214) had no response after vaccination. The difference between responders and non-responders regarding the disease subgroups was highly significant (*p* < 0.0001). Considering the subgroup with negative responses, in myeloid neoplasms, patients with Philadelphia chromosome negative (Ph −) myeloproliferative neoplasms (MPN) had the highest proportion of negative seroconversion (*n* = 6/13), followed by patients with myelodysplastic syndromes (MDS, *n* = 5/13). In the group including all patients with chronic myeloid leukemia (CML), 100/101 (99%) had a positive vaccine response (see Tables 2, 3, and 4). The association between response and entity for the subgroup of myeloid neoplasms revealed to be significant (*p* = 0.0011). A negative antibody titer was measured in one patient with CML and JAK2 V617F-positive PMF. This patient received combined treatment with ruxolitinib and bosutinib.

**Table 2:** Malignant and other hematological disease entities and negative antibody response

Hematological disease entities	Total cohort of hematological disease		With negative antibody response		
	**Cohort (*n* = 373)**	**Percentage of entities group**	**Group (*n* = 56)**	**Percentage of total group of this entity**	**Percentage of entities group**
**Myeloid neoplasms**	**214**	**100%**	**13**	**6.1%**	**100%**
MPN	76	36%	6	7.9%	46%
CML	101	47%	1	1.0%	7.7%
MDS	21	10%	5	24%	39%
AML	16	7.5%	1	6.3%	7.7%
**Lymphoid neoplasm**	**124**	**100%**	**39**	**31%**	**100%**
Indolent NHL	60	48%	25	42%	64%
Aggressive NHL	18	15%	8	44%	21%
Multiple myeloma	31	25%	4	13%	10%
Hodgkin lymphoma	9	7.3%	1	11%	2.6%
ALL	6	4.8%	1	17%	2.6%
**Other**	**35**	**100%**	**4**	**11%**	**100%**
Autoimmune	26	74%	4	15%	100%
Benign	9	26%	0	0%	0%

*MPN*, myeloproliferative neoplasm; *CML*, chronic myeloid leukemia; *MDS*, myelodysplastic syndrome; *AML*, acute myeloid leukemia; *NHL*, non-Hodgkin lymphoma; *ALL*, acute lymphatic lymphoma

**Table 3:** Patients in active treatment and negative antibody response

Hematological disease entities	In active treatment			With negative antibody response			
	**Total (*n* = 229)**	**Percentage of hematological disease (% of 373)**	**Percentage of entities group**	**Group (*n* = 39)**	**Percentage of in treatment entities total group**	**Percentage of total group with neg. AB** **(% of 56)**	**Percentage of entities group**
**Myeloid neoplasms**	**152**	**41%**	**100%**	**10**	**6.6%**	**18%**	**100%**
MPN	64	17%	42%	5	7.8%	8.9%	50%
CML	74	20%	49%	1	1.3%	1.8%	10%
MDS	10	2.7%	6.6%	3	30%	5.4%	30%
AML	4	1.0%	2.6%	1	25%	1.8%	
**Lymphoid neoplasm**	**56**	**15%**	**100%**	**25**	**45%**	**45%**	**100%**
Indolent NHL	28	7.5%	50%	17	61%	30%	68%
Aggressive NHL	6	1.6%	11%	5	83%	8.9%	20%
Multiple myeloma	16	4.3%	29%	3	19%	5.4%	12%
Hodgkin lymphoma	5	1.3%	8.9%	0	0%	0%	0%
ALL	1	0.3%	1.8%	0	0%	0%	0%
**Other**	**21**	**5.6%**	**100%**	**4**	**19%**	**7.1%**	**100%**
Autoimmune	17	4.6%	81%	4	24%	7.1%	100%
Benign	4	1.0%	19%	0	0%	0%	0%
**Total**	**229**	**61%**	**100%**	**39**	**17%**	**70%**	**100%**

*MPN*, myeloproliferative neoplasm; *CML*, chronic myeloid leukemia; *MDS*, myelodysplastic syndrome; *AML*, acute myeloid leukemia; *NHL*, non-Hodgkin lymphoma; *ALL*, acute lymphatic lymphoma

**Table 4:** Patients previously treated/treatment naïve and negative antibody response

Hematological disease entities	Previously treated/treatment naïve (39%)			With negative antibody response			
	**Total (*n* = 144)**	**Percentage of hematological disease (% of 373)**	**Percentage of entities group**	**Group (*n* = 17)**	**Percentage of non-treatment entities total group**	**Percentage of total group with neg. AB** **(% of 56)**	**Percentage of entities group**
**Myeloid neoplasms**	**62**	**17%**	**100%**	**3**	**4.8%**	**5.4%**	**100%**
MPN	12	3.2%	19%	1	8.3%	1.8%	33%
CML	27	7.2%	44%	0	0%	0%	0%
MDS	11	2.9%	18%	2	18%	3.6%	67%
AML	12	3.2%	19%	0	0%	0%	0%
**Lymphoid neoplasm**	**68**	**18%**	**100%**	**14**	**21%**	**25%**	**100%**
Indolent NHL	32	8.6%	47%	8	25%	14%	57%
Aggressive NHL	12	3.2%	18%	3	25%	5.4%	21%
Multiple myeloma	15	4.0%	22%	1	6.7%	1.8%	7.1%
Hodgkin lymphoma	4	1.1%	5.9%	1	25%	1.8%	7.1%
ALL	5	1.3%	7.3%	1	20%	1.8%	7.1%
**Other**	**14**	**3.7%**	**100%**	**0**	**0%**	**0%**	**100%**
Autoimmune	9	2.4%	64%	0	0%	0%	0%
Benign	5	1.3%	36%	0	0%	0%	0%
**Total**	**144**	**39%**	**100%**	**17**	**12%**	**30%**	**100%**

*MPN*, myeloproliferative neoplasm; *CML*, chronic myeloid leukemia; *MDS*, myelodysplastic syndrome; *AML*, acute myeloid leukemia; *NHL*, non-Hodgkin lymphoma; *ALL*, acute lymphatic lymphoma

### Impact of gender and age

A total of 38/56 patients (68%) with negative antibody titer were male, and 18/56 patients (32%) were female. In patients with positive response, 171/317 (54%) were male. This difference slightly failed to reach statistical significance (*p* = 0.0531). Older age was associated with a negative antibody titer. Medians for birth year were 1946 and 1958 for patients with negative or positive response, respectively (*p* < 0.0001). In particular, negative titers were found in elderly patients with aggressive and indolent NHL (Tables 2, 3, and 4).

### Impact of therapy

In the group with negative seroconversion, 39/56 (70%) of patients were on active therapy whereas 17/56 (30%) were previously treated (≥ 3 months before vaccination) or treatment naïve (low-grade NHL *n* = 8, high-grade NHL *n* = 3, MDS *n* = 2, MM *n* = 1, M. Hodgkin *n* = 1, MPN *n* = 1, ALL *n* = 1). In the group with positive responses, 190/311 were on active therapy (*p* = 0.224).

Independent of the treatment status, lymphoid disorders (indolent and aggressive) were predominant in this subgroup. In patients with indolent NHL, negative antibody response was present on different treatment regimens: 7/8 patients were on BTK inhibitor (ibrutinib or acalabrutinib), and 5/8 patients were on rituximab-based chemotherapy. Moreover, 8/32 patients with indolent NHL without any therapy the last 3 months before 1st vaccination had no seroconversion. In the group of aggressive NHL, 5/8 patients with no antibody response were on active therapy.

A total of 28/373 patients were treated with ruxolitinib and most of them (*n* = 24) were diagnosed with MPN. Four/24 patients had a negative antibody response (age: 69–88 years), whereas 20/24 patients had a measurable positive seroconversion (age: 42–86 years). Nevertheless, antibody titers below 100 U/mL were detected in 9/20 patients with a positive immune response. In MDS patients, no correlation was found between specific therapy or age and negative vaccination response.

Twenty-five/373 patients (with AML (*n* = 10), MDS (*n* = 5), CML (*n* = 5), MPN (*n* = 2), ALL (*n* = 2), and low-grade NHL (*n* = 1)) received an allogeneic stem cell transplantation prior to vaccination. In 3/25 patients, no seroconversion was measured with a range from 6 months to 8 years between transplantation and vaccination. Patients without response were on ruxolitinib treatment due to graft versus host disease and hemophagocytosis or transplantation was performed recently (*n* = 1).

### Univariable and multivariable analyses

**Fig. 1:**
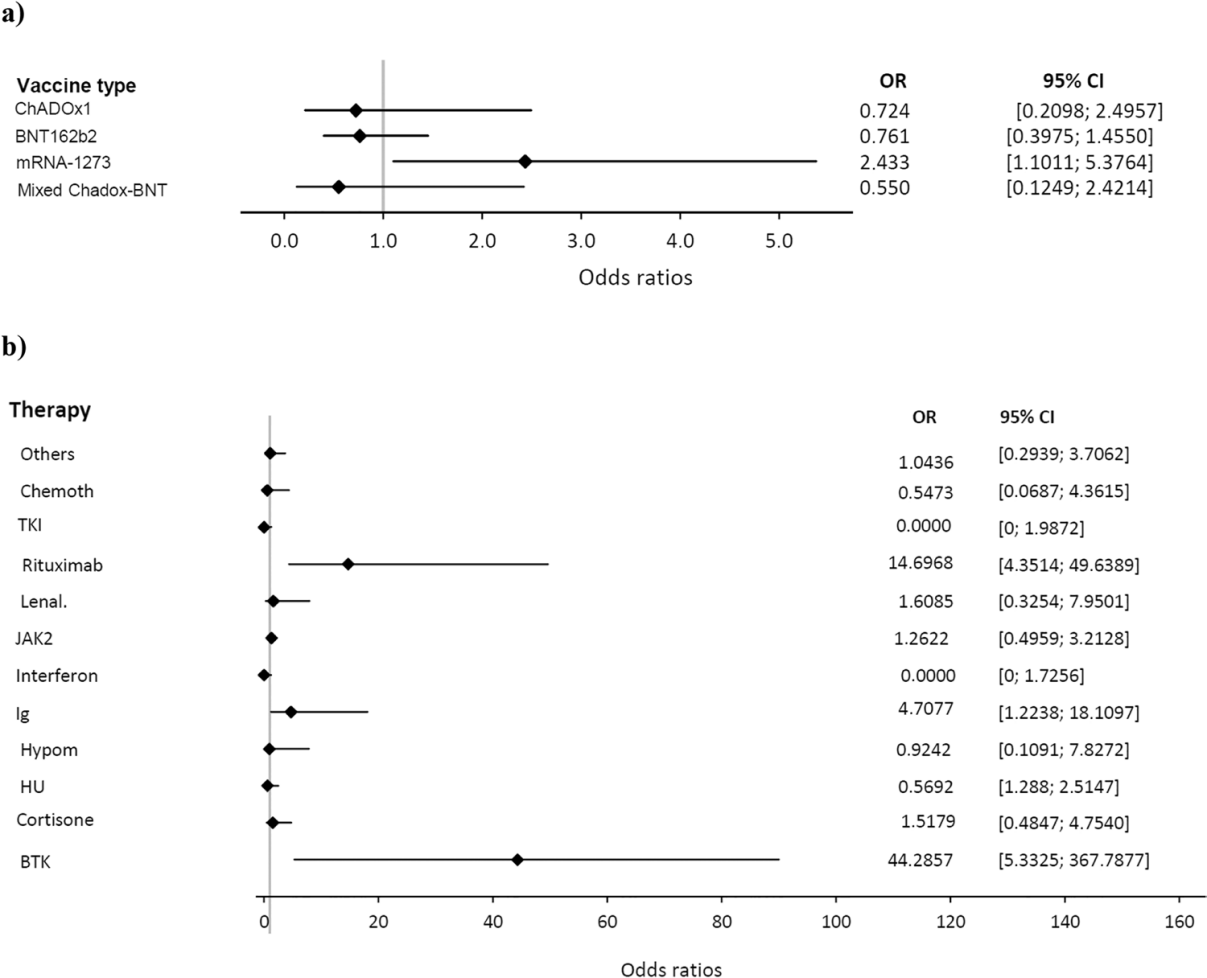
Forest plots of univariable logistic regression models with odds ratio (OR) and confidence intervals demonstrating risk for negative vaccination response correlating with **a** the vaccine used and **b** treatments. Legend: CI, confidence interval; Chemoth, chemotherapy; TKI, tyrosine kinase inhibitors; Lenal., lenalidomide; JAK2, Jak2-inhibitors; Ig, immunglobulin substitution; Hypom., hypomethylating agents; HU, hydroxyurea; BTK, BTK inhibitors

In univariable analyses, the following factors were significantly associated with negative vaccination response: older age (*p* < 0.0001), indolent NHL (*p* < 0.0001), aggressive NHL (*p* = 0.0021), and mRNA-1273 (Moderna) vaccine (*p* = 0.0241; s. Figure 1). Patients with CML were significantly less likely to have negative response. These patients were on TKI treatment or in treatment-free remission [20]. MPN diagnosis slightly failed to reach statistical significance (*p* < 0.0001 and *p* = 0.0515), respectively.

**Fig. 2:**
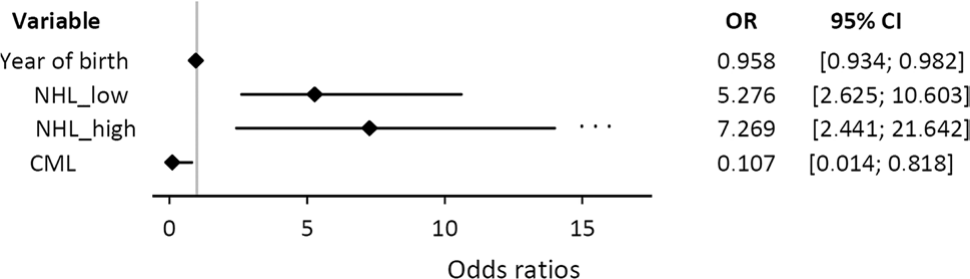
Forest plots of the multivariable logistic regression model with odds ratio (OR) and confidence intervals demonstrating risk for negative vaccination response. Legend: CI, confidence interval; NHL_low, indolent non-Hodgkin lymphoma; NHL_high, aggressive non-Hodgkin lymphoma; CML, chronic myeloid leukemia

Odds ratios and confidence intervals for some relevant factors are presented in Fig. 2. Therapies associated significantly with no antibody response were rituximab (OR = 14.7), BTK inhibitors (OR = 44.3), and immunoglobulin substitution (OR = 4.7).

In the multivariable logistic regression analysis using the forward-selection method, diagnosis of indolent (*p* < 0.0001, odds ratio (OR) = 1.6) and aggressive NHL (*p* = 0.0004, OR = 2.0) was found to increase the risk for negative vaccination response. On the other hand, protective impact was found for CML (*p* = 0.0312, OR = 0.105; s. Figure 2). Regarding the year of birth, the risk for a negative vaccination response decreased with each year (*p* = 0.0008, OR = 0.956) indicating that older people (with a lower year of birth) have a higher risk.

Taking out the CML patient group as very good responders, factors influencing vaccination status did not change.

Time after vaccination, gender and being on active treatment in general had no significant impact on the seroconversion rate after vaccination.

## Discussion

Our data of this single-center study comprise one of the biggest well-characterized patient group and correspond to a real-world cohort in a hematology ambulatory setting which enabled us to identify important factors influencing COVID-19 vaccination success in patients with hematological disorders.

The central finding of our study is that 99% of patients with CML had a positive seroconversion after the 2nd COVID vaccination. This confirms the results from a recently published smaller study [21].

Furthermore, in our cohort active treatment per se did not significantly correlate with negative vaccination response as reported in other studies summarized in a recent meta-analysis [7]. A possible explanation might be that our patient cohort included a substantial number of patients with negative seroconversion which have been treated earlier or were treatment naïve. Patients with different disorders including mainly indolent NHL belonged to this group indicating that not only patients on active treatment should be screened for antibody response.

In accordance to the abovementioned meta-analysis, patients diagnosed with lymphoid neoplasms (especially indolent and aggressive NHL) were at higher risk for negative seroconversion compared to patients with other hematological diseases [7]. However, any patients with other hematological disorders, especially MPN and MDS patients, had a high risk for negative vaccination result.

As described in the healthy population, older age was correlated with a negative antibody response to COVID-19 vaccination [22]. Age was related to negative antibody titers of all entities in a multivariable analysis. Regarding the year of birth, the risk for a negative vaccination response increased with each year.

In the univariable analysis, vaccination with mRNA-1273 (Moderna) correlated with negative antibody response. In contrast, in the multivariable analysis with addition of further influencing factors, no significance was discernible. As the number of patients vaccinated with Moderna was small and included predominantly NHL patients, no conclusion can be drawn out of this result.

As found in other studies, target-specific therapies were correlated with negative antibody response [9, 10, 23]. Therapies significantly associated with a negative seroconversion were BTK inhibitors like ibrutinib, acalabrutinib, rituximab, and immunoglobulin substitution. TKI, interferon, lenalidomide, hypomethylating agents, and other treatments did not demonstrate significance in our analysis. Patients with ivIG therapy represent a heterogenous disease group (patients with B-CLL, indolent NHL, multiple myeloma, etc.). Despite the fact that ivIG therapy was associated with reduced vaccination response, the therapy per se is unlikely the determining factor. Rather, in these patients, the underlying disease may have an impact on seroconversion.

However, most of the patients with MPN and no seroconversion received ruxolitinib. In patients with MDS, there was no correlation between specific therapy and negative response.

Allogeneic stem cell transplantation was not a negative predictor in our cohort as most patients were in longer follow-up after the procedure, which correlates with one recent published study [24]. The patients with negative response received treatment after complications.

Latest studies have shown a stronger decrease of vaccination response over the time associated with higher age [13, 16, 17] and antibody titers decrease every month [16, 17]. Our preliminary results suggest that vaccine titers may decrease much more rapidly in patients with hematologic diseases compared to the general population. To clarify this, more systemic prospective data on a possible higher decline over time especially in hematologic patients are needed.

In this context, another important aspect is the influence of antibody titers lower than 250 U/mL or even below 100U/mL. In our cohort, a substantial amount (20%) of patients were measured with titers below 100U/mL. Titers above 0.8 U/mL are considered positive but it is still unclear what level is representative for a sufficient protection. Thus, a classification of the effectiveness is important in order to be able to counteract with specific measures at an early stage, e.g., prioritization and earlier booster vaccination as already recommended for patients with immunodeficiency [9, 23]. Other vaccination strategies [25] or treatments with monoclonal antibodies pre-/post expositional might be essential for these patients [8, 26].

Neutralizing antibodies can prevent the host cell infection by binding to the spike protein. Although our study did not examine the cellular immune response, there is evidence that measurement of S-protein antibody levels correlates with neutralizing antibody titers as it was demonstrated in several studies [13, 14, 19, 27–30].

Since new variants of SARS-CoV-2 have emerged, the question is whether neutralizing antibodies can also capture viral variants and whether this correlates with antibody titers after vaccination [15]. It may be due to mutations in the RBD, as is the case with Omicron, that protection by neutralizing antibodies is not sufficient. Therefore, it is equally important to pay attention to the T cell response after vaccination in this context [31].

It seems like T cells show protection against severe COVID-19 and it appears that patients develop a cellular immune response after vaccination despite little or no seroconversion [32].

Vaccinated individuals do have a T cell immunity to the SARS-CoV-2 Omicron variant, potentially balancing the lack of neutralizing antibodies in preventing or limiting severe COVID-19 [31]. Booster vaccinations could be important to further restore cross-neutralization by antibodies [32]. However, patients with measurable antibody titers may still not be protected against infection because their antibodies may not be neutralizing as described above.

In summary, our data defined important predictive factors for negative antibody response after COVID-19 vaccination in patients with hematological diseases. Negative seroconversion was significantly correlated with lymphoid diseases, older age, and therapy with BTK inhibitors, rituximab, and immunoglobulin substitution. In addition, ruxolitinib therapy was an important negative predictor in MPN patients. Active antitumoral therapy per se was not a negative predictive factor. Vaccination of CML patients was significantly associated with positive seroconversion.

In conclusion, these data help to identify patients at risk for negative seroconversion after COVID-19 vaccination and to adapt further clinical decisions to prevent these patients more effectively from COVID-19.

## Data Availability

The datasets generated and/or analyzed during the current study are available from the corresponding author on reasonable request.
